# Supplementary figures and images for: Cuticular collagens mediate cross-kingdom predator–prey interactions between trapping fungi and nematodes
Source: PLoS Biol. 2025 Jul 1;23(7):e3003178. doi: 10.1371/journal.pbio.3003178 (PMC12237271; doi:10.1371/journal.pbio.3003178)

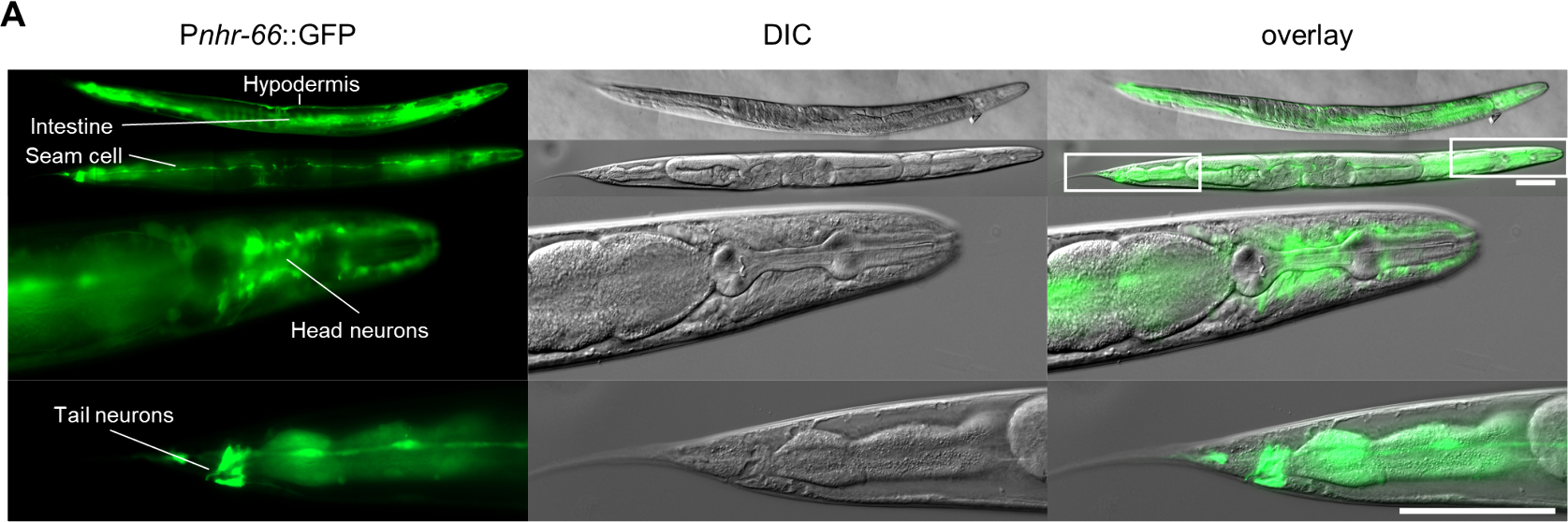

Supplement: S1 Fig — White boxes indicate regions shown at higher magnification in subsequent panels (scale bar, 100 µm). (TIF) [file pbio.3003178.s010.tif]

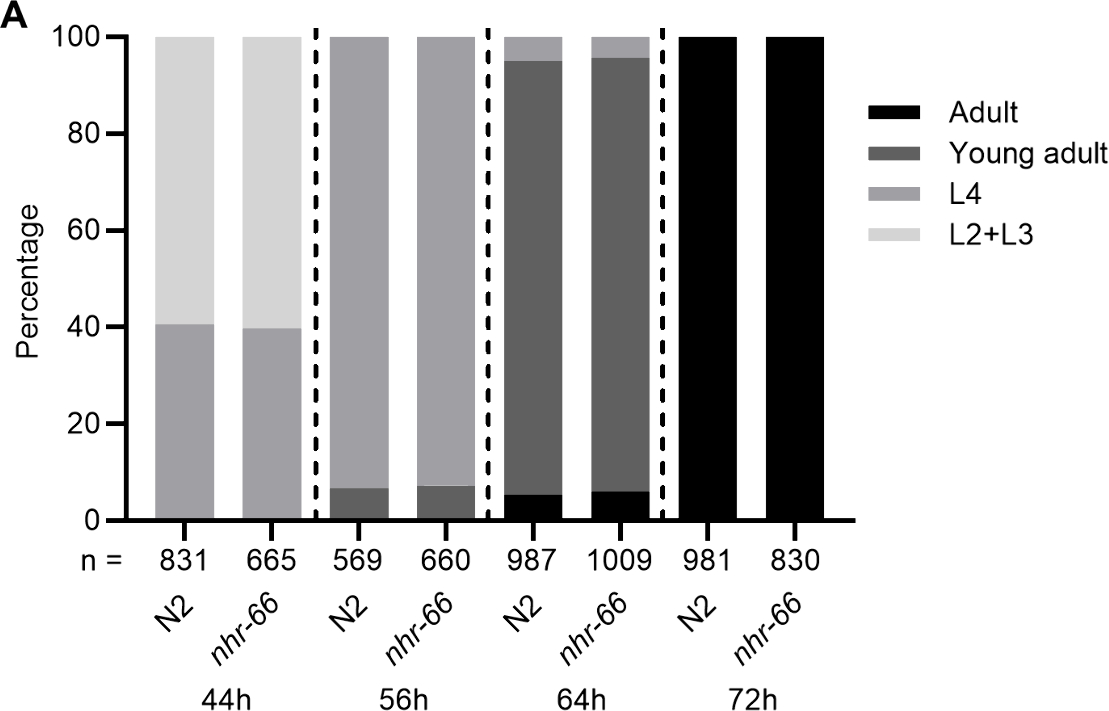

Supplement: S2 Fig — (n is shown below the x-axis). The data underlying this figure can be found in S6 Data. (TIF) [file pbio.3003178.s011.tif]

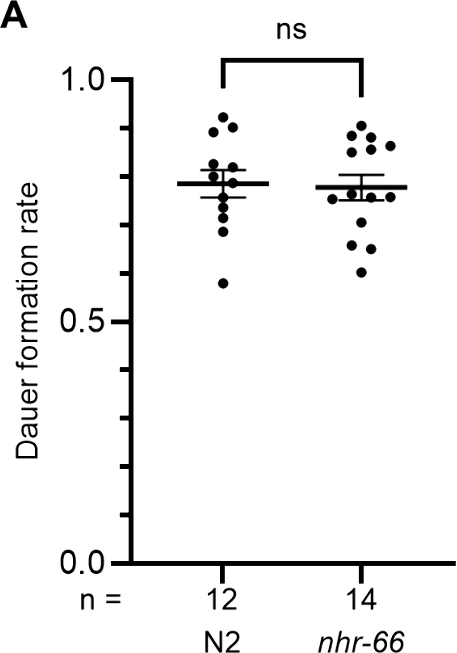

Supplement: S3 Fig — (mean ± SEM, n is shown below the x-axis, two-tailed unpaired Student t test). The data underlying this figure can be found in S6 Data. (TIF) [file pbio.3003178.s012.tif]

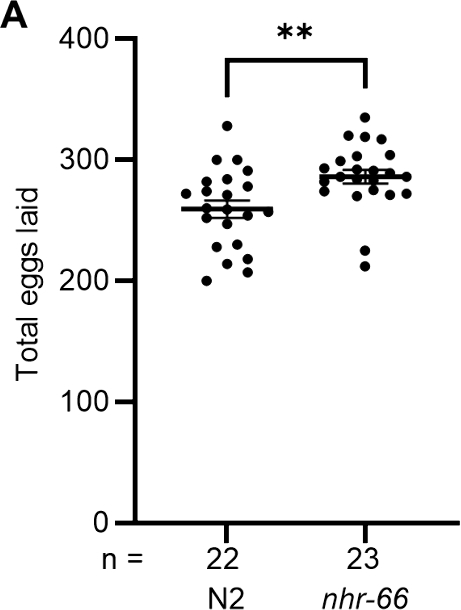

Supplement: S4 Fig — (mean ± SEM, n is shown below the x-axis, two-tailed unpaired Student t test). The data underlying this figure can be found in S6 Data. (TIF) [file pbio.3003178.s013.tif]

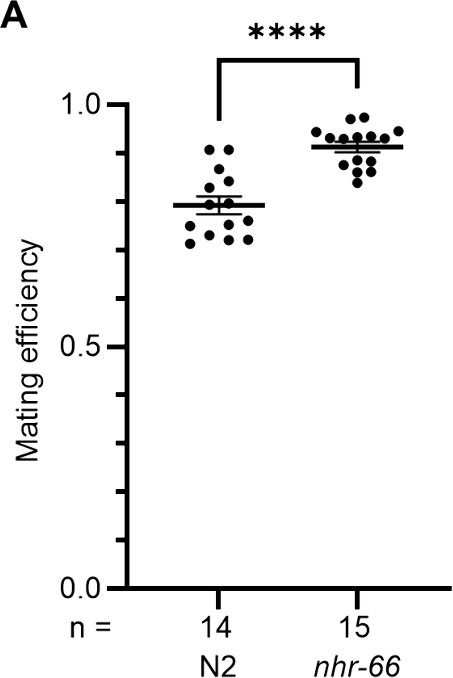

Supplement: S5 Fig — (mean ± SEM, n is shown below the x-axis, two-tailed unpaired Student t test). The data underlying this figure can be found in S6 Data. (TIF) [file pbio.3003178.s014.tif]
